# Real-world epidemiology and management of patients with rheumatoid arthritis treated with methotrexate in France: a decade after methotrexate first recommendations

**DOI:** 10.1016/j.ero.2026.03.003

**Published:** 2026-04-03

**Authors:** René-Marc Flipo, Denis Jullien, Louis Chillotti, Charlotte Cancalon, Mélanie Goguillot, Françoise Bugnard, Stève Bénard, Philippe Gard, Alison Ducreux, Mathias Rouard, Pascale Mermet, Bruno Fautrel

**Affiliations:** 1 Rheumatology, Lille University Hospital, Lille, France; 2 Dermatology, Lyon University Hospital–HCL, Lyon, France; 3 stève consultants–a Cytel company, Oullins, France; 4 Medac France, Lyon, France; 5 Rheumatology, Paris University Hospital–APHP, Paris, France

## Abstract

**Objectives:**

Methotrexate (MTX) is the primary systemic first-line treatment of rheumatoid arthritis (RA). Following the treat-to-target principle, MTX dose and administration route should be optimised before considering advanced therapies (biologic/targeted synthetic disease-modifying antirheumatic drugs [b/tsDMARDs]), notably in case of insufficient response or intolerance. This study examined patients with RA initiating MTX, assessed MTX optimisation before b/tsDMARD escalation, and evaluated b/tsDMARD economic impact.

**Methods:**

This observational cohort study used claims data from the French national insurance database, identifying patients with RA (International Classification of Diseases–10th revision M05/M06) who began MTX treatment between January 1, 2016 and December 31, 2021. Among them, patients initiating MTX in 2016-2017 were followed through the end of the study or death. MTX persistence and b/tsDMARD escalation were analysed using the Kaplan-Meier estimator. Costs were described for a 1-year period before and after b/tsDMARD escalation.

**Results:**

Between 2016 and 2021, 52,462 patients with RA initiated MTX (≈8700/y), mostly orally (79.1%). Mean (SD) age was 60.8 (14.3) years, with 71.0% of women. Two-year MTX persistence was 73.7% (73.0%-74.4%), increasing by ≈20 points in the case of form switch (from 66.8% to 83.8%). Also, 17.5% (16.9%-18.1%) of patients escalated to b/tsDMARD. Among the 3582 patients who escalated to b/tsDMARD, 1121 (31.3%) were treated with oral MTX and were not optimised with subcutaneous MTX before b/tsDMARD. Mean (SD) costs increased from €6218 (€12,259) in the year before to €13,131 (€10,408) the year after b/tsDMARDs initiation.

**Conclusions:**

Despite a high persistence, many patients escalated to b/tsDMARDs without complete optimisation. Full optimisation of MTX in terms of dose and administration route could be one therapeutic means of delaying escalation to b/tsDMARD, thus preserving future treatment options.

## INTRODUCTION

### Rheumatoid arthritis: pathophysiology and management

Rheumatoid arthritis (RA) is a chronic inflammatory disease characterised by pain and progressive stiffness that leads to joint destruction, especially in the wrists, hands, and fingers. RA affects between 200,000 and 300,000 patients in France, with a prevalence of 0.33%, and typically manifests among women aged between 35 and 55 years [[1], [2], [3]].

According to the latest French and European guidelines, methotrexate (MTX) is the primary first-line systemic treatment in cases of active RA. Other conventional synthetic disease-modifying antirheumatic drugs (csDMARDs—leflunomide and sulfasalazine) should be considered in cases of MTX contraindication. MTX can be associated with analgesics, nonsteroidal anti-inflammatory drugs (NSAIDs), as well as with low-dose corticosteroids during the first 6 months of treatment. MTX should be initiated at a weekly dose of 10 to 15 mg and quickly (1-3 months) optimised to a dose up to 25 or 30 mg, notably based on MTX effectiveness and tolerance. If MTX proves ineffective or poorly tolerated, advanced targeted therapies (biologic/targeted synthetic disease-modifying antirheumatic drugs [b/tsDMARDs]) may be introduced [[4], [5], [6], [7]]. With weekly doses exceeding 15 to 20 mg, MTX digestive absorption is saturated, leading to a plateau in efficacy, as well as an increased risk of digestive adverse events. The subcutaneous (SC) form, available in France since 2015, can be used in relay and helps reach higher dosages [4,8,9]. Finally, tolerance remains one of the key challenges in MTX optimisation, with almost 80% of patients experiencing adverse events (notably gastrointestinal manifestations—≈30%), and around 10% of patients discontinuing MTX due to tolerance concerns [10]. Some studies have highlighted the better tolerance of SC MTX compared with the oral form, notably concerning gastrointestinal adverse effects [11,12], but this impact remains highly debated [13,14].

### Rationale and objectives

Although MTX is still primarily administered orally, the SC form offers potential efficacy advantages by overcoming absorption limitations and potentially improving digestive tolerance [15]. In addition to potentially increasing MTX persistence and adherence, optimisation of MTX in dose and pharmaceutical form may also be associated with a delayed need for escalation to b/tsDMARDs and, consequently, prevent the healthcare costs increase associated with biotherapy [[16], [17], [18]]. However, real-world studies exploring MTX optimisation strategies in RA remain limited, particularly since these practice changes.

This study aimed to (i) characterise patients with RA initiating MTX, (ii) describe MTX treatment patterns before b/tsDMARD escalation, and (iii) assess the economic implications of b/tsDMARD escalation.

## METHODS

### Design and patient selection

This study was an observational, national, real-world analysis using claims data from the French National Healthcare Insurance System (Système national des données de santé - SNDS). SNDS includes all reimbursed inpatient and outpatient services for nearly 99% of French residents. Data in the SNDS are pseudonymised and provide detailed patient sociodemographic information (eg, age, sex (male/female), city of residence, vital status) alongside extensive records of healthcare utilisation. This includes reimbursed ambulatory healthcare services (eg, medical and paramedical care, care settings, laboratory tests, imaging, medical devices, and dispensed drugs) as well as services delivered within healthcare facilities (eg, type of facility, admissions and discharges, diagnoses, procedures, and costly drugs billed outside the diagnosis-related group [DRG] system) [19,20]. SNDS data from 2012 to 2021 (the latest year available at the time of the study) were extracted.

Patients with RA who initiated MTX between January 1, 2016 and December 31, 2021 were selected to describe their main characteristics at MTX initiation. Patients who began treatment in 2016 or 2017 were followed from MTX initiation until the end of the study period (December 31, 2021) or death, whichever occurred first. This provided a sufficient medical history to ensure MTX initiation, and allowed assessment of previous treatments used (4-year medical history), as well as long-term use of RA treatments, with up to 6 years of data after MTX initiation.

RA was identified as having at least one hospitalisation or long-term disease status with a RA diagnosis (International Classification of Diseases–10th revision codes M05-M06) [21]. MTX initiation was determined based on the absence of any DMARD dispensation within a 5-year historical period before the first MTX dispensation. Patients with a history of conditions for which MTX is also indicated—such as lupus (L93, M32), systemic sclerosis (M34), myositis (M60), ankylosing spondylitis (M45), psoriasis (L40), or psoriatic arthritis (M07)—were excluded.

### Outcomes and variables

Sociodemographic characteristics (age, sex, area of residence, and free-of-charge coverage [22]), clinical characteristics identified over the 5 years preceding MTX initiation (eg, Charlson comorbidity index [23], hypertension, myocardial infarction, treated dyslipidaemia, diabetes, cancer, inflammatory bowel disease, and chronic respiratory diseases), along with treatment history (NSAIDs, analgesics, and corticosteroids), were described at MTX initiation, both overall and by MTX form (oral/SC).

Treatment patterns were assessed from MTX initiation to the study’s end, death, or b/tsDMARDs introduction, whichever came first. These patterns included MTX form at initiation (oral/SC), form switching, codispensations, MTX discontinuation, MTX injectable dose adjustments, and b/tsDMARD escalation.

#### Discontinuation

In order to consider the real-world nature of the data and the fact that SNDS only covers dispensations, MTX was considered as discontinued if no dispensation was observed for a period of 3 consecutive months. In this case, based on MTX dispensation practices in France, dispensations were considered to cover 1 month of treatment: SC MTX is mostly dispensed in 4-unit packages, covering 1 month, whereas oral MTX is available in boxes of 10 tablets of 10 mg and 24 tablets of 2.5 mg, potentially covering 4 to 8 weeks. Hence, a conservative approach was preferred, and the discontinuation date was set as 1 month after the last dispensation observed, in order to cover the last package received. Also, potential stockpiling was not taken into consideration. Of note, the discontinuation motive is not available in the SNDS.

#### MTX optimisation

As the assessment of MTX oral dose is based on the type and number of packages dispensed, it is subjected to informational bias. However, the recommended dose increase (≈25 mg/wk) before considering b/tsDMARDs cannot be reached with oral MTX. Hence, MTX optimisation was assessed among patients who escalated to b/tsDMARDs, and was derived based on current guidelines, using the MTX form, and SC MTX dose. MTX was considered as fully optimised if b/tsDMARDs escalation was preceded by injectable MTX at a dose ≥15 mg.

#### Impact on healthcare resource use

Healthcare resource use (HCRU) and associated costs were evaluated over a 1-year period before and after b/tsDMARDs escalation in patients on b/tsDMARDs. Relevant HCRUs included RA medications, medical visits, paramedical care, hospitalisations, laboratory and imaging tests, and medical transport. Costs were detailed overall and by HCRU type.

Patient selection criteria and outcome identification were based on validated codes and algorithms, available in the Supplementary Material.

### Statistical analyses

MTX discontinuation and b/tsDMARDs escalation were analysed using the Kaplan-Meier estimator. Patients were censored at the study’s end or death, and at b/tsDMARDs escalation for MTX discontinuation. MTX optimisation patterns were displayed among patients who escalated to biotherapy as Sankey diagrams. Finally, the factors associated with b/tsDMARDs escalation were assessed among patients who received a single form of MTX. A Cox regression model was developed, adjusting for age, MTX form at initiation, region of residence, and whether the first MTX prescription was issued in a hospital setting.

Healthcare costs reimbursed by the French health insurance were reported by HCRU type in €2021.

### Ethics

The study was conducted in accordance with the International Society for Pharmacoepidemiology guidelines and the principles outlined in the Declaration of Helsinki. As part of the regulatory submission for claims studies utilising French SNDS data, this study received approval from the French Ethics and Scientific Committee [24] on January 13, 2022 (dossier 3925449); as well as from the French Data Protection Agency [25], dossier MLD/MTY/AR223740 pursuant to Article 13 of the law and Resolution No. 2019-021 of February 28, 2019 (authorisation 922053).

In addition, an independent scientific committee was involved from the protocol development stage to ensure the scientific and methodological rigour as well as the clinical relevance of the study and its findings.

## RESULTS

Among the 215,952 patients who initiated MTX treatment between 2016 and 2021, 58,445 (27.1%) had RA.

Of these, 5,352 patients met noninclusion criteria, resulting in a final study population of 52,462 (24.3%). This resulted in an average of 8,743 patients with RA initiating MTX annually. This number increased from 7,747 in 2016 to 9,014 in 2021, with a peak at 9,960 in 2019.

### Patients' characteristics

The sex ratio was 2.4, with 37,248 (71.0%) female. The mean (SD) age at MTX initiation was 60.8 (14.3) years, with 9,136 (17.4%) patients aged 75 years or older. Most patients (77.5%, n = 38,067) resided in urban areas. The mean Charlson comorbidity score at MTX initiation was 5.0 (2.5), with no patient presenting a score of 0. The most common comorbidity was arterial hypertension, observed in 20,930 (39.9%) patients. Other significant comorbidities included chronic respiratory diseases (24.7%), dyslipidaemia (22.3%), depression (19.0%), and diabetes (11.7%). More than 80% of patients had a history of treatment with corticosteroids (83.8%, n = 43,977) or NSAIDs (86.1%, n = 45,185). No notable differences were observed between the MTX pharmaceutical forms (Table).

**Table:** Sociodemographic and clinical characteristics—study population (N = 52,462)

Characteristics	Study population initiating with oral MTX (n = 39,881)	Study population initiating with SC MTX (n = 12,581)	Study population (N = 52,462)
Year of MTX initiations (n)			
2016	6,254	1,493	7,747
2017	6,135	1,786	7,921
2018	6,661	2,132	8,793
2019	7,386	2,574	9,960
2020	6,749	2,278	9,027
2021	6,696	2,318	9,014
Age			
Mean (SD)	60.8 (14.3)	60.7 (14.3)	60.8 (14.3)
Median (IQR)	62.0 (51.0-71.0)	62.0 (51.0-71.0)	62.0 (51.0-71.0)
Sex			
Sex ratio	2.5	2.5	2.5
Female, n (%)	28,355 (71.1)	8,895 (70.7)	37,248 (71.0)
Free-of-charge coverage, n (%)			
Yes	3,589 (9.0)	1,246 (10.7)	4960 (9.5)
Area of residence, n (%)			
Urban	30,948 (77.6)	9,675 (76.9)	38,067 (77.5)
Rural	8,933 (22.4)	2,906 (23.1)	11,082 (22.5)
Charlson comorbidity index			
Mean (SD)	4.9 (2.5)	5.1 (2.5)	5.0 (2.5)
Median (IQR)	5.0 (3.0-6.0)	5.0 (3.0-7.0)	5.0 (3.0-6.0)
Comorbidities of interest, n (%)			
Atherosclerosis	971 (2.4)	362 (2.9)	1333 (2.5)
Arterial hypertension	15,778 (39.6)	5,152 (41.0)	20,930 (39.9)
Myocardial infarction	581 (1.5)	189 (1.5)	770 (1.5)
Dyslipidaemia	9,047 (22.7)	2,674 (21.3)	11,721 (22.3)
Diabetes	4,449 (11.2)	1,676 (13.3)	6,125 (11.7)
Hepatic failure	564 (1.4)	252 (2.0)	816 (1.6)
Depression	7,454 (18.7)	2,521 (20.0)	9,975 (19.0)
Cancers	3,288 (8.2)	1,081 (8.6)	4,369 (8.3)
Chronic respiratory diseases	9,578 (24.0)	3,359 (26.7)	12,937 (24.7)
Inflammatory bowel diseases	221 (0.6)	96 (0.8)	317 (0.6)
History of treatments of interest, n (%)			
NSAIDs	34,691 (87.0)	10,494 (83.4)	45,185 (86.1)
Analgesics	37,640 (94.4)	11,436 (90.9)	49,076 (93.5)
Corticosteroids	33,689 (84.5)	10,288 (81.8)	43,977 (83.8)

MTX, methotrexate; NSAID, nonsteroidal anti-inflammatory drug; SC, subcutaneous.

Free-of-charge coverage represents the full coverage of medical expenses for patients with limited resources. All codes and algorithms are available in the Supplementary Material.

Within this cohort, 15,668 patients initiated their treatment in 2016 or 2017 and were included in the analysis of MTX treatment patterns, the factors associated with treatment escalation, and the costs.

### MTX treatment patterns

#### MTX initiation and switch

MTX was primarily initiated in the oral form, with 79.1% (n = 12,389) of patients starting treatment this way, whereas 20.9% (n = 3,279) initiated with the SC form. Although the oral dose at initiation cannot be assessed precisely in SNDS, the SC dose at initiation was 15.2 (3.8) mg/wk.

Among patients who began treatment with oral MTX, 5,043 (40.7%) switched to SC MTX during follow-up. The median (IQR) time to switch was 195.0 (91.0-462.0) days, or approximately 6.5 months. Of those who switched to SC MTX, 1,357 (26.9%) subsequently returned to oral MTX after a median of 620.0 (327.0-1057.0) days, equivalent to around 20.7 months on SC MTX.

Conversely, among patients who initiated treatment with SC MTX, 722 (22.0%) switched to oral MTX during follow-up. The median (IQR) time to switch in this group was 390.0 (170.0-827.0) days, ie, roughly 13 months. Of these patients, 267 (37.0%) switched back to SC MTX after a median of 539.0 (257.0-1010.0) days on oral MTX, which corresponds to approximately 17.6 months.

Subsequent switches between MTX forms were not further analysed in this study due to the small number of patients involved.

#### MTX discontinuation

Based on the Kaplan-Meier estimator, 73.7% (73.0%-74.4%) of patients were still receiving MTX treatment after 24 months of follow-up, and 55.8% (54.9%-56.7%) at 60 months. This rate was 71.2% (69.5%-72.8%) among patients who initiated with SC MTX, and 74.4% (73.6%-75.2%) for those starting with the oral form (Fig 1A).

**Figure 1:**
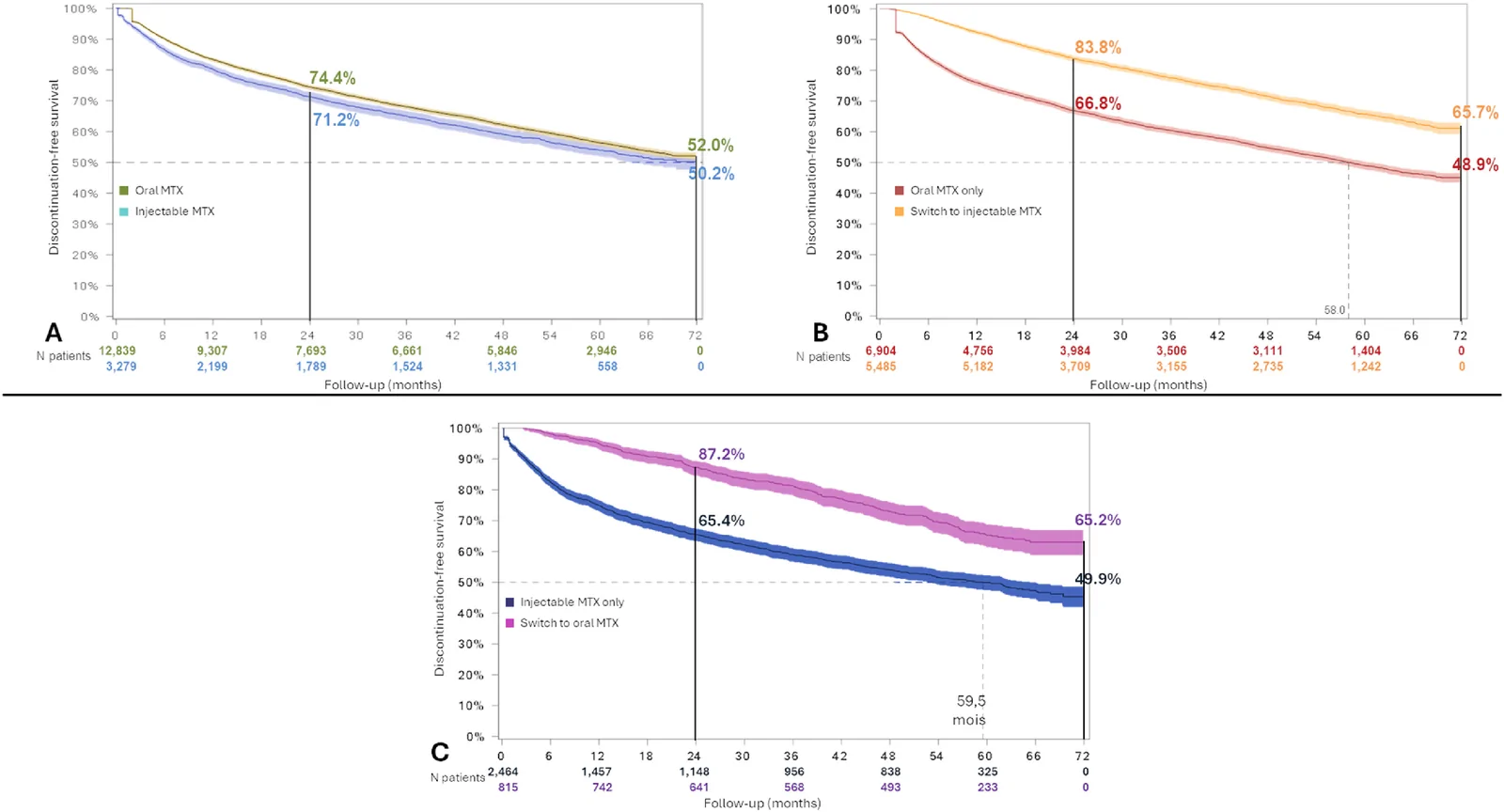
Discontinuation-free survival functions after MTX initiation according to: (A) initial MTX form, (B) presence of a switch to SC MTX among patients initiating with oral MTX, (C) presence of a switch to oral MTX among patients initiating with SC MTX—Kaplan-Meier estimator. MTX, methotrexate; SC, subcutaneous.

Among the 6,904 patients treated exclusively with oral MTX and the 5,485 patients who switched to the SC form, those who switched exhibited greater persistence. At 24 months, the proportion of patients still receiving MTX was 66.8% (65.7%-68.0%) for those treated with the oral form only, compared with 83.8% (82.7%-84.8%) for patients using both oral and SC forms. At 60 months, these rates were 48.9% (47.6%-50.3%) and 65.7% (64.2%-67.2%), respectively (Fig 1B).

A similar trend was observed among the 2,464 patients with RA treated exclusively with SC MTX and the 815 patients who switched to the oral form. At 24 months, the proportion of patients still receiving MTX was 65.4% (63.3%-67.4%) for those treated with SC MTX only, compared with 87.2% (84.6%-89.4%) for patients using both SC and oral forms. At 60 months, these rates were 49.9% (47.5%-52.3%) and 65.2% (61.4%-68.8%), respectively (Fig 1C).

#### MTX optimisation patterns before b/tsDMARDs

As oral MTX is dispensed in boxes of 2.5 mg (24 units) or 10 mg (10 units) tablets, a significant informational bias made it impossible to precisely assess MTX dosage for the oral form. The results showed a mean and median dose of 15 mg/wk, whatever the timepoint considered. Hence, the dose was only presented for patients with SC MTX. Among the 2,464 patients treated only with SC MTX, the mean (SD) MTX doses at 3, 6, and 12 months were 15.7 (4.1), 16.3 (4.3), and 16.3 (4.6) mg/wk, respectively. The proportion of patients receiving a dose of less than 15 mg/wk was 18.6% at 3 months, 14.4% at 6 months, and 15.3% at 12 months. Among the 988 patients treated only with SC MTX who eventually escalated to b/tsDMARDs, the last MTX dose dispensed before escalation was, on average, 17.5 (4.4) mg/wk. Of these patients, 13.7% were still receiving a dose of less than 15 mg/wk, whereas 47.2% had a dose of at least 20 mg/wk.

Based on the Kaplan-Meier estimator, almost 75% of patients did not escalate to b/tsDMARD after 5 years. The rate was 26.2% (25.5%-26.9%) patients escalated to a b/tsDMARD at 60 months. Patients who initiated with SC MTX (23.7% [22.3%-25.1%]) tended to escalate earlier than those who initiated with the oral form (15.9% [15.2%-16.5%]) (Fig. 2).

**Figure 2:**
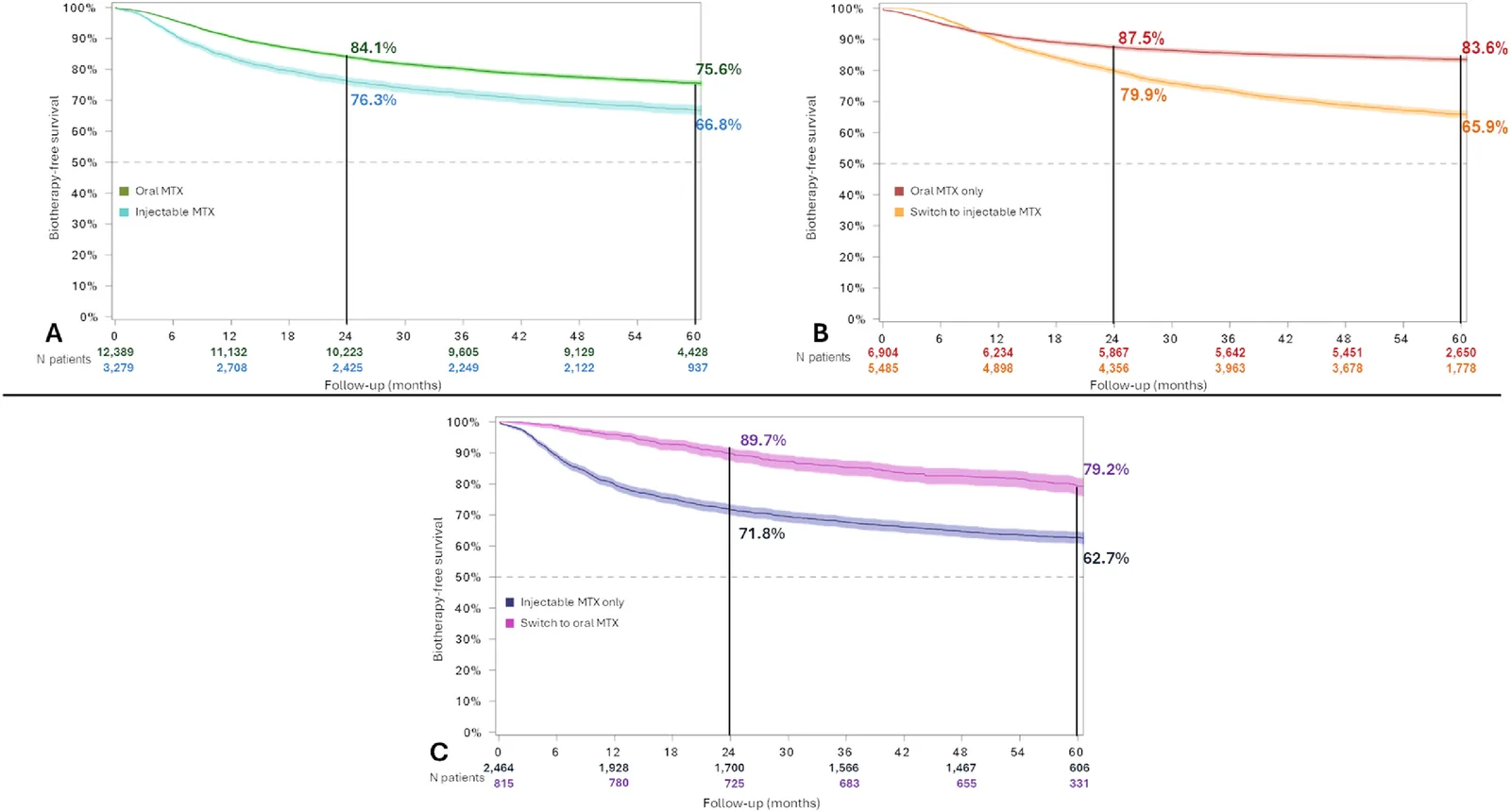
b/tsDMARDs-free survival functions after MTX initiation according to: (A) initial MTX form, (B) presence of a switch to SC MTX among patients initiating with oral MTX, (C) presence of a switch to oral MTX among patients initiating with SC MTX—Kaplan-Meier estimator. b/tsDMARD, biologic/targeted synthetic disease-modifying antirheumatic drug; MTX, methotrexate; SC, subcutaneous.

MTX optimisation consisted in the presence of an SC MTX dose ≥15 mg before b/tsDMARD escalation and was assessed among the 3,582 patients who escalated to b/tsDMARD. Of them, 2,604 (72.7%) started with oral MTX, 270 (7.5%) with SC MTX at a dose <15 mg, and 708 (19.8%) with SC MTX at a dose ≥15 mg. Among the 2,604 patients who initiated with oral MTX, 1,121 (43.0%) escalated directly to b/tsDMARD, ie, without SC MTX optimisation. The remaining 1,483 (57.0%) patients switched to SC MTX, mostly (91.8%, n = 1361) at a dose ≥15 mg, before escalating to b/tsDMARD, highlighting a complete MTX optimisation.

Among the 270 patients who initiated with SC MTX <15 mg, 204 (75.6%) increased the MTX dose before switching to b/tsDMARD, showing a complete MTX optimisation; 56 (20.7%) escalated directly to b/tsDMARD; and 10 (3.7%) switched to oral MTX.

Finally, among the 708 patients who initiated with SC MTX ≥15 mg, 569 (80.4%) escalated directly to b/tsDMARD, showing a complete MTX optimisation; 82 (30.4%) decreased SC MTX dose, and 57 (21.1%) switched to oral MTX.

Of note, only 195 (5.4%) patients had another switch in MTX dose/form before b/tsDMARD escalation, 73 of whom finished with SC MTX ≥15 mg.

Overall, 2,283 (63.7%) patients presented complete MTX optimisation before escalating to b/tsDMARDs, implying that more than one-third (36.3%, n = 1,299) of patients escalated to b/tsDMARD without complete optimisation (Fig 3).

**Figure 3:**
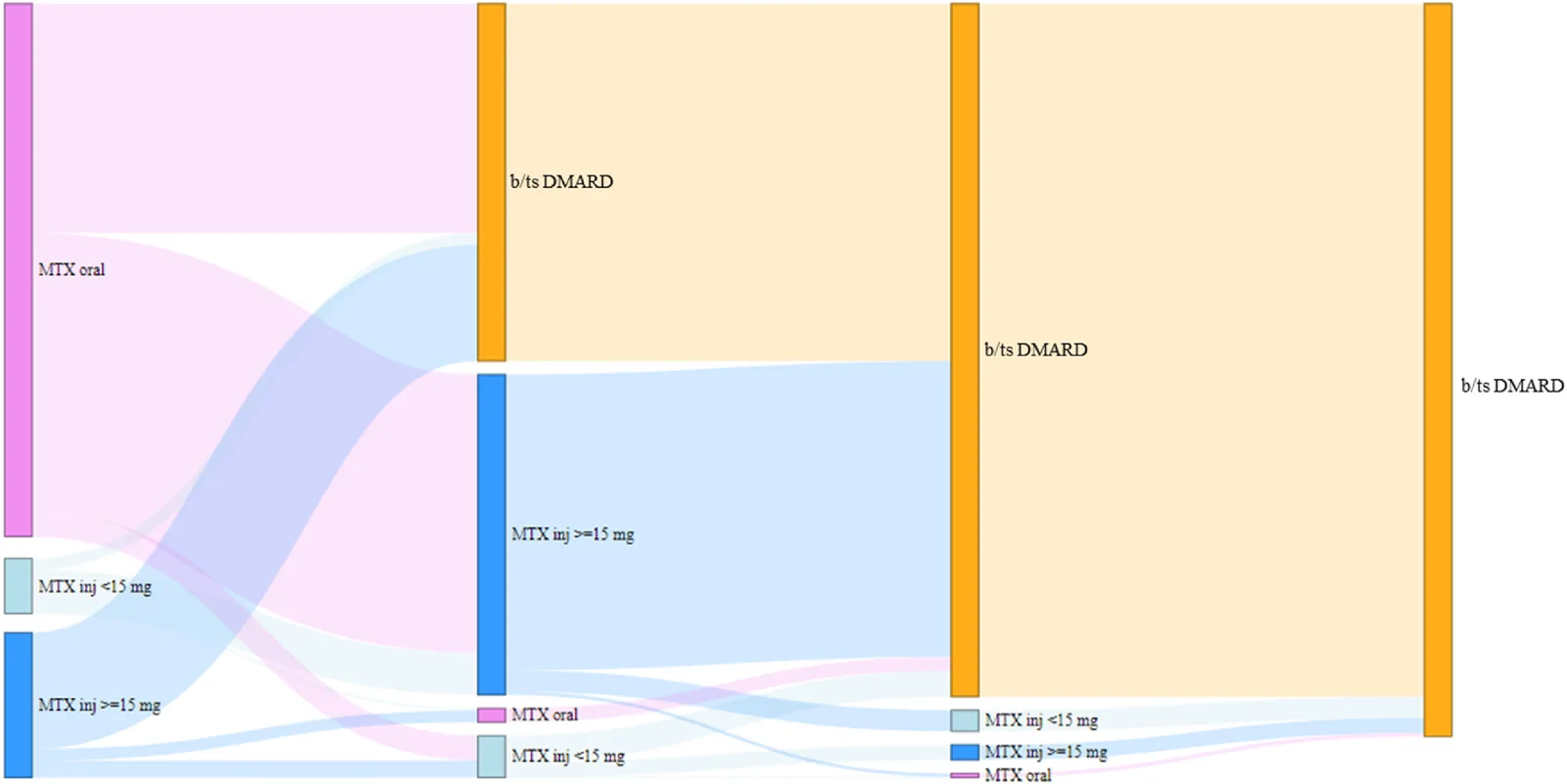
MTX dose and optimisation pattern before b/tsDMARDs escalation among patients with a b/tsDMARDs dispensation over follow-up (n = 3582)—Sankey representation. b/tsDMARD, biologic/targeted synthetic disease-modifying antirheumatic drug; MTX, methotrexate.

### Factors associated with b/tsDMARD escalation

The Cox model assessing factors associated with b/tsDMARD escalation was developed using data from 9,923 patients treated with a single form of MTX during follow-up (7,346 on oral MTX and 2,577 on SC MTX). All tested variables of interest—age, region of residence, MTX form, and initiation settings—were significant (Fig 4). A notable interaction was observed between MTX form and initiation settings (outpatient vs hospital).

**Figure 4:**
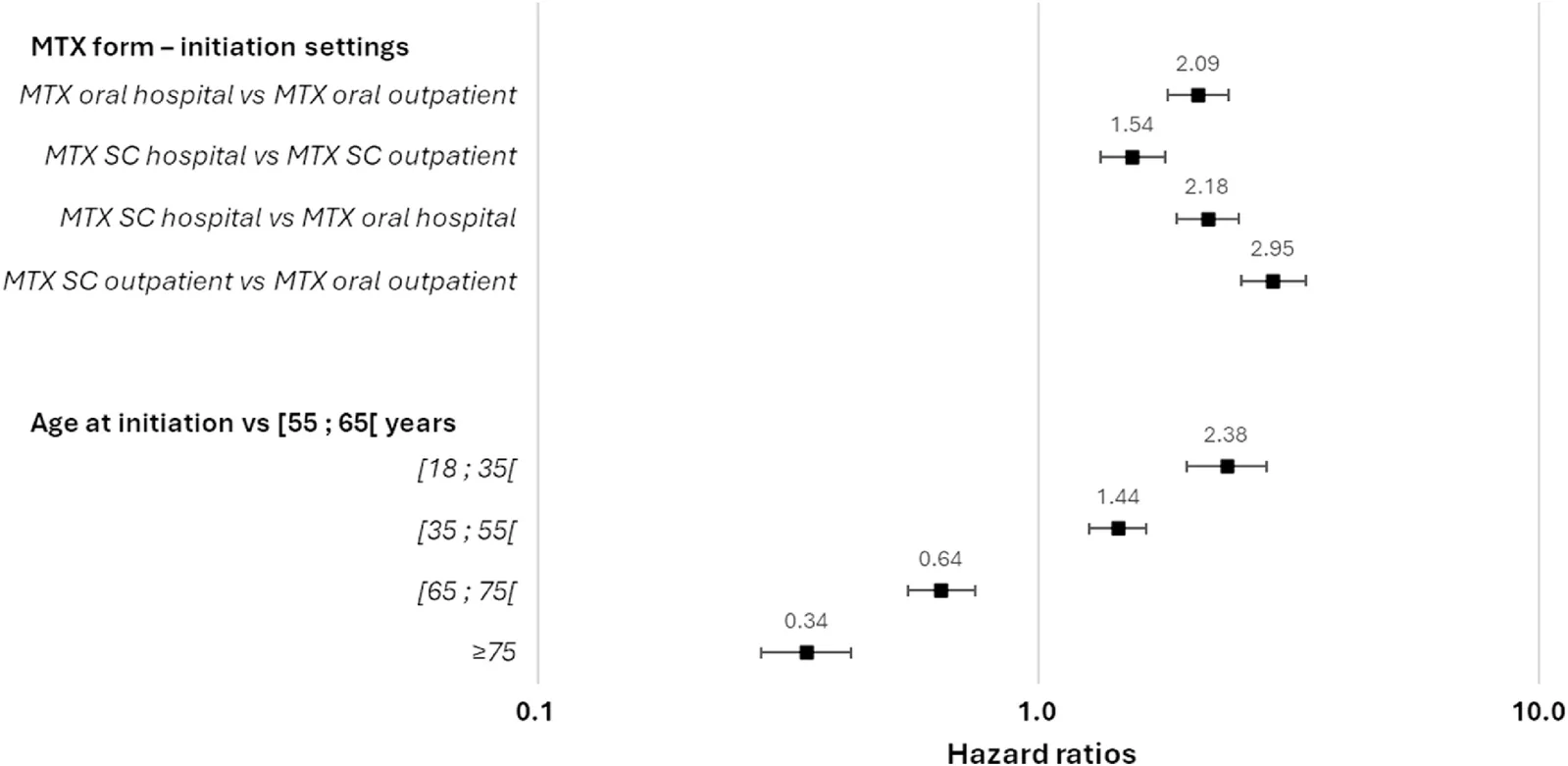
Hazard ratios from the Cox model on time from MTX initiation to b/tsDMARDs escalation among patients treated only with 1 form (N = 9923). b/tsDMARD, biologic/targeted synthetic disease-modifying antirheumatic drug; MTX, methotrexate; SC, subcutaneous.

Overall, age was the primary factor associated with b/tsDMARD escalation in our model, with the likelihood of escalation decreasing as the age at MTX initiation increased. Specifically, compared with the 55 to 65 years subgroup, corresponding to the mean age at MTX initiation, hazard ratios (HRs—95% CI) were 0.64 (0.55-0.75) in the 65 to 75 years subgroup, and 0.34 (0.28-0.42) in the ≥75 years subgroup, indicating a lower probability of escalation. Conversely, in the 35 to 55 years subgroup, the HR was 1.44 (1.27-1.64), and 2.38 (1.98-2.86) in the 18 to 35 year subgroup, reflecting a higher probability of escalation to b/tsDMARD.

Regarding MTX form and initiation settings (outpatient vs hospital), treatment with SC MTX was associated with a higher risk of escalation to biologic therapy compared with oral MTX, with HRs of 2.95 (2.54-3.42) for outpatient initiation and 2.18 (1.89-2.51) for hospital initiation. Conversely, initiation in hospital was associated with a higher risk of escalation compared with outpatient initiation, with HRs of 1.54 (1.33-1.80) for SC MTX and 2.09 (1.82-2.40) for oral MTX, respectively (Fig 4).

### Costs associated with b/tsDMARDs escalation

HCRU and costs were described among the 3,664 patients treated with b/tsDMARDs and presenting at least 12 months of data before and after b/tsDMARD escalation.

When comparing the year before and after b/tsDMARD escalation, the proportion (95%CI) of patients with at least 1 medical visit significantly decreased from 90.3% (89.3%-91.3%) to 80.3% (79.0%-81.6%), notably for general practitioner visits (from 48.4% [46.7%-50.0%] to 18.8% [17.6%-20.1%]). A similar trend was observed for hospitalisations, which decreased from 59.3% (57.7%-60.9%) to 48.7% (47.1%-50.4%), and imaging exams, which decreased from 89.3% (88.3%-90.3%) to 62.1% (60.5%-63.6%) (Fig 5A).

**Figure 5:**
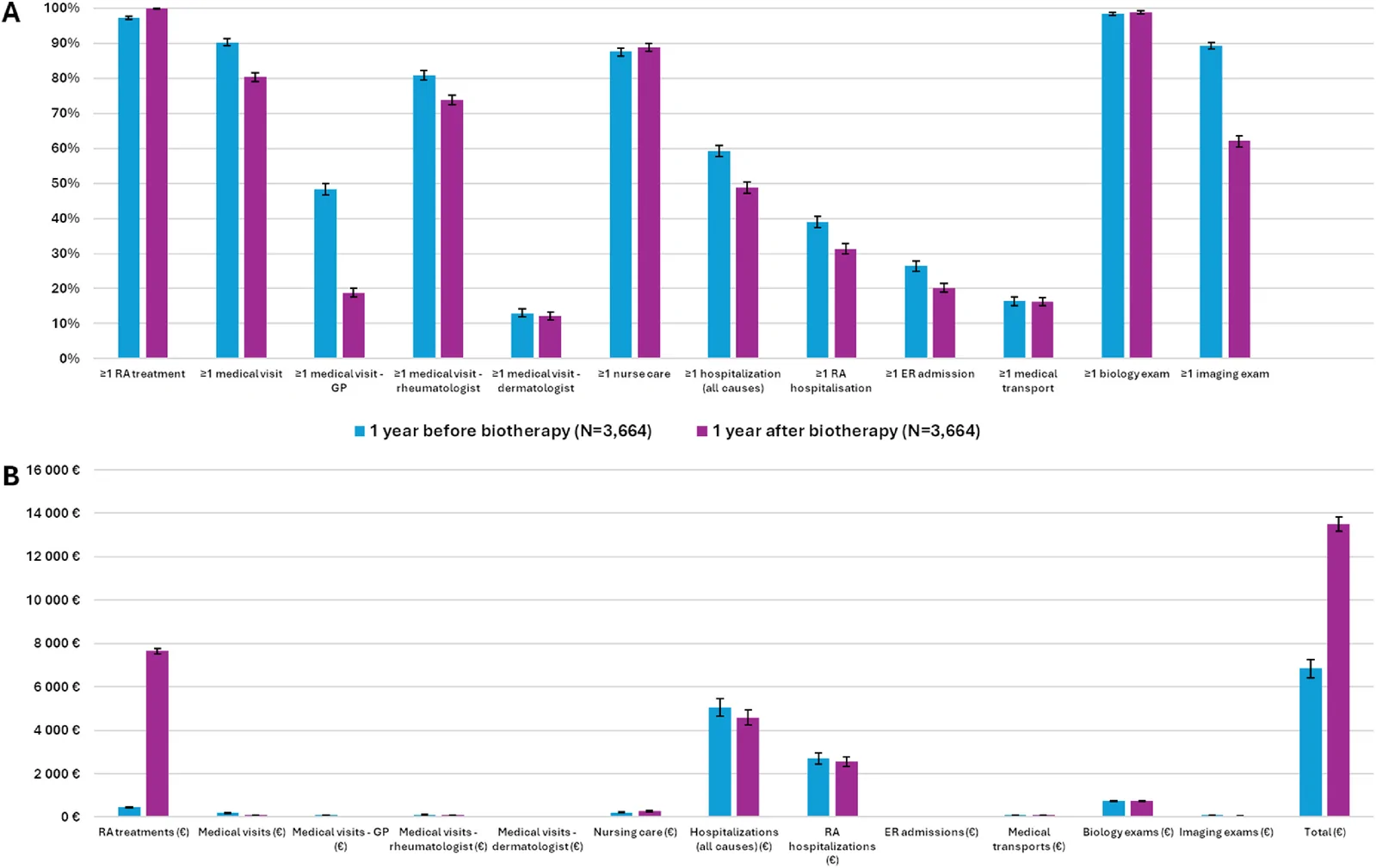
Proportion of patients with HCRU of interest (A) and mean (SD) costs per HCRU of interest (B) 1 year before and 1 year after b/tsDMARDs escalation among patients treated with b/tsDMARDs with at least 1 year of follow-up after b/tsDMARDs escalation (n = 3664). b/tsDMARD, biologic/targeted synthetic disease-modifying antirheumatic drug; ER, emergency room; GP: general practitioner; HCRU, healthcare resource use; RA, rheumatoid arthritis; SC, subcutaneous.

During this period, the mean (SD) total cost increased from €6,218 (€12,259) to €13,131 (€10,408). RA medications became the first expense item, increasing from 6.9% (€432) to 57.7% (€7,572) of the total costs. Hospitalisations represented 35.3% (€4,636) of the costs before biologic therapy, compared with 33.7% (€4,437) after b/tsDMARD escalation. Medical visits accounted for 2.3% (€148) and 0.6% (€82) before and after b/tsDMARD escalation, respectively (Fig 5B).

## DISCUSSION

Between 2016 and 2021, nearly 50,000 patients with RA initiated treatment with MTX, averaging around 8000 patients per year. According to several recent French studies, over 300,000 patients are affected by RA, with approximately 13,000 initiating MTX annually [2]. Sociodemographic and clinical characteristics were consistent with the literature [2,3,7].

Although most patients initiated treatment with oral MTX, the proportion of patients starting with SC MTX slightly increased over time. Although a rise in RA severity cannot be ruled out, a more likely explanation may be a progressive change in prescribing practices towards higher doses and the use of SC MTX, mirroring the evolution of the French recommendations. For instance, following the METOP study in 2017 for psoriasis, a notable change in prescription patterns towards SC MTX was observed [13,26]. As previously noted, SC MTX provides better bioavailability [8] and allows for higher-dose escalation while potentially decreasing the gastrointestinal adverse events commonly observed with oral MTX. This is, however, still being debated in the literature, with studies advocating for an equivalence in adverse event rates and severity between pharmaceutical forms, whereas others emphasise lower risks with SC MTX, and even a decrease in adverse events with switch to SC form [[11], [12], [13], [14]]. Several studies have indicated that these adverse events were among the most frequent causes of oral MTX discontinuation [16,17], and that around 10% of discontinuation may be caused by adverse events [10].

This study demonstrated MTX great persistence over time, with over 70% still treated at 24 months, and more than half at 60 months, which is significantly higher than that of b/tsDMARDs—roughly 50% at 2 years [27]. Notably, MTX optimisation through switching to another formulation significantly increased overall persistence by approximately 20% at 24 months (≈85% with switch vs ≈65% without). Of note, alternative reasons for longer persistence cannot be ruled out. Notably, the therapeutic target, codefined by the patient and practitioner, could directly impact the duration a patient stays under MTX. Based on residual disease activity acceptability, patients may refuse a dose increase or keep MTX for a longer period. Also, the treat-to-target strategy recommends reaching low disease activity during the first months, which could be longer for some patients (treatment inertia), for whom MTX persistence will be artificially increased to reach the target [28,29].

Two main therapeutic optimisation strategies were identified in this study. First, the switch from oral to SC MTX observed in 40.7% of patients. This switch occurred approximately 6.5 months after initiation, aligning with current guidelines for MTX optimisation. This change may reflect an increase in MTX dosage and a transition to the SC form to enhance bioavailability or to minimise gastrointestinal adverse events [4,6,30]. Second, the switch from SC to oral MTX was seen in 22.0% of patients. This switch may indicate a period of reduced clinical repercussions during which the MTX dose could have been decreased as part of the treat-to-target approach, or an effort to enhance patient comfort in response to injection fatigue [31,32].

Consistent with treatment persistence, this study found that 60 months after initiating MTX, nearly 75% of patients did not escalate to b/tsDMARDs. This finding aligns with data from the French ESPOIR cohort, which reported a 10-year escalation rate of 30% and depict the current guidelines on MTX and b/tsDMARD escalation [5,[33], [34], [35]].

With this context, the analysis of MTX optimisation patterns revealed that most patients escalated to b/tsDMARDs before achieving MTX optimisation (i.e., being treated with at a dose of ≥15 mg with SC MTX before b/tsDMARDs). Notably, nearly half of the patients who initiated treatment with oral MTX escalated directly to b/tsDMARDs. Considering the guideline-driven definition of MTX optimisation used in this study, this pattern may suggest that the MTX dose may not have been fully optimised before b/tsDMARDs, particularly considering the limitations of oral MTX absorption at higher doses (15-20 mg) [8,16,[36], [37], [38]]. Nonetheless, current practices for MTX optimisation heavily rely on RA clinical targets (notably disease activity score [DAS28] and imaging findings), disease impact on quality of life, treatment tolerance, and patient preferences [4]. These key components are not captured in the SNDS, resulting in information bias that should be considered in the interpretation. Treatment tolerance appears to be a major determinant of MTX optimisation, with approximately 80% of patients experiencing adverse events, around 30% of which are gastrointestinal [10]. In such cases, guidelines recommend switching to another csDMARD for nonsevere disease or escalating to a b/tsDMARD otherwise. In both situations, these changes typically occur before reaching the standard MTX target dose of 25 mg, that is , before full optimisation. Another similar scenario involves patients who initially achieve full MTX optimisation but subsequently develop adverse events, leading to dose reduction and treatment escalation.

For patients starting with SC MTX, this choice may have stemmed from the necessity for a high initial dose, potentially due to elevated disease activity, or it could reflect patient preference or practitioner prescribing habits. Concerning dosage calculations, only those for SC MTX could be accurately assessed, as 1 unit consistently corresponds to 1 weekly dose. In contrast, oral dispensations, although thoroughly identified, exhibited significant variability in terms of the number of boxes dispensed and the dosages administered, as well as the intervals between dispensations. This variability led to results that were often difficult to interpret, particularly due to the incremental dose escalation, typically in minimum increments of 2.5 mg [8,9]. For this reason, patients with oral MTX directly escalating to b/tsDMARD (ie, without switch to SC) were considered as nonoptimised. As specified above, the guideline-derived definition of MTX optimisation could possibly exclude some patients for whom an imperative clinical situation (not identifiable in the SNDS database) forced the practitioner to escalate to a second line without optimising MTX from initial oral posology.

Overall, age at MTX emerged as the primary factor associated with subsequent escalation to b/tsDMARDs, with younger patients demonstrating a higher likelihood of receiving b/tsDMARDs. This reduced probability of switching to b/tsDMARDs among older patients has been observed in several cohort studies. Potential explanations for this trend include therapeutic de-escalation initiated by either the physician or the patient, often driven by considerations of comfort or the presence of contraindications [35,39]. In addition, the interaction observed between MTX formulation at initiation and the setting of treatment initiation (outpatient vs hospital) may be partly explained by channelling bias. Patients initiating MTX in a hospital setting are more likely to present with more severe disease, warranting higher initial doses and potentially earlier treatment escalation. Moreover, hospital‑based physicians may be more familiar with, or more inclined to prescribe, SC MTX. Other clinical factors not captured in the SNDS may also influence escalation to b/tsDMARDs.

This study indicated that the initiation of b/tsDMARDs resulted in a slight reduction in the proportion of patients requiring hospitalisations or medical visits, along with a decreasing trend in overall healthcare costs. However, the initiation of b/tsDMARDs was associated with a significant increase in total costs, rising from €6,800 in the year preceding initiation to €13,500 in the year following it. This increase, primarily attributed to the cost of the treatment itself, does not appear to be offset by reductions in other healthcare utilisations, such as medical visits and hospitalisations. Of note, the use of b/tsDMARDs is associated with more severe and expensive disease forms, probably not sufficiently tackled by csDMARDs. Also, the current decrease in advanced therapy costs due to biosimilar availability is not exhaustively captured in the period covered by this study. Hence, although b/tsDMARDs are associated with a notable increase in treatment cost, it is not possible to draw conclusion on the efficiency (cost-effectiveness) of MTX, nor of b/tsDMARDs with this observational study. The impact of these therapies should be assessed in light of potential improvements in patients’ quality of life, particularly among populations that may have been inadequately controlled with MTX alone.

Finally, this study relies on claims data and describes MTX use between 2016 and 2021. This period covers several updates to RA treatment guidelines and prescribing practices. To assess the potential impact of these changes on our findings, we compared the 3 guideline updates closest to our study period (2016, 2019, and 2022). Regardless of the guideline considered, MTX consistently remained the cornerstone of first-line systemic therapy in RA and was not recommended solely in cases of contraindication or intolerance. Although the maximum recommended dose varied between 25 and 30 mg/wk, all guidelines emphasised the importance of rapid dose escalation to achieve the target dose. By contrast, the main differences across guidelines related to glucocorticoid use, the selection of alternative csDMARDs, and prioritisation among b/tsDMARDs in cases of treatment escalation [4,6,40]. Consequently, from an MTX-centred perspective, RA management remained relatively consistent over time, reinforcing the role of MTX as the keystone of first-line RA therapy and lending support to the robustness of our study findings.

SNDS exhaustively gathers healthcare reimbursements of French residents, notably hospitalisations, medical and paramedical care, and treatment received, making it a suitable tool for epidemiological research, assessment of treatment use, and of economic burden at a population level. This study provides a comprehensive and accurate overview of the epidemiology and use of MTX for RA in France and highlights the fact that some patients may be escalating to b/tsDMARD too early, and could benefit from MTX for a longer time before b/tsDMARDs escalation by optimising MTX dose and pharmaceutical form (route of administration). There are, so far, few data available on patients with RA outcome and their continued treatment after diagnosis of RA and initiation of MTX. The Canadian (CATCH) and English (ERAS) cohorts can only provide partial answers [41,42]. This analysis of SNDS data brings important additional information.

## Conclusion

This study showed that MTX is the cornerstone of first-line therapy for RA, with over 8,000 patients initiating this treatment annually. More than half of the patients remained on MTX 5 years after initiation. This persistence rate increased by 20 percentage points among those who underwent therapeutic optimisation (change in MTX form), either for efficacy increase or for comfort/tolerance—compared with those who did not switch formulations. In addition to adaptations for clinical reasons, this optimisation in dose and pharmaceutical form may be associated with a delayed need for escalation to biologic therapies. Over half of patients did not require such escalation 5 years postinitiation. However, 43% of patients who began oral treatment escalated to b/tsDMARDs without optimisation, as derived from current guidelines (ie, MTX dose increase and switch to SC form to bypass oral absorption saturation). Hence, patients may be escalating to b/tsDMARD too early, and could benefit from MTX for a longer time before b/tsDMARDs escalation by optimising MTX dose and form. Widespread adoption of MTX optimisation before b/tsDMARDs escalation may be associated with significant cost reductions, considering the difference in cost between MTX and b/tsDMARDs, and assuming an equivalent clinical response. Ultimately, optimising MTX makes it possible to significantly postpone the use of second-line treatments, thus preserving the armamentarium of biologic therapies for the future if necessary.

## Editor disclosure

The peer review process did not involve Editorial Board Member Bruno Fautrel, and the editorial decision-making was led by editors who were not involved in the creation of this manuscript

## Competing interests

R-MF received honoraria from AbbVie, Amgen, BMS, Celltrion, Galapagos, Janssen, Lilly, Medac, MSD, Nordic, Pfizer, and Sandoz. DJ received honoraria from AbbVie, Almirall, Amgen, BIF, BMS, Janssen, Leo, Lilly, Medac, Pfizer, Sanofi, and UCB. LC, MG, FB, SB, and CC are employees of stève consultants, which has a research contract with Medac France. PG, MR, AD, and PM are employees of Medac France. BF received honoraria from AbbVie, Alfasigma, Biocon, BMS, Celltrion, Fresenius Kabi, Lilly, Medac, Nordic, Novartis, Owkin, Pfizer, and Swedish Orphan Biovitrum.

## Appendix Supplementary materials

Image, application 1

## Footnotes

Supplementary material associated with this article can be found in the online version at doi:10.1016/j.ero.2026.03.003.
