# Supplementary material for: Real-world epidemiology and management of patients with rheumatoid arthritis treated with methotrexate in France: a decade after methotrexate first recommendations
Source: EULAR Rheumatol Open. 2026 Apr 3;2(3):100155. doi: 10.1016/j.ero.2026.03.003 (PMC13650332; doi:10.1016/j.ero.2026.03.003)
Supplement: Supplementary file 1 [file mmc1.docx]

**Real-World Epidemiology and Management of Patients with Rheumatoid Arthritis Treated with Methotrexate in France: a Decade after Methotrexate First Recommendations**

*SNDS*

Codes and Algorithms

# Identification of patients initiating MTX

Table 1. Identification of patients initiating MTX

Patients with all of the three following criteria :

- Newly treated with MTX between 01JAN15 and 31DEC16
- Aged >=18 at initiation (first MTX dispensation)
- With no previous MTX dispensation in the 5 years before initiation

**Codes in annex**

Source : (1)

# Identification of patients with RA initiating MTX

Table 2. Identification of patients with RA initiating MTX

**Patients avec une polyarthrite rhumatoïde (PR)**

**Inclusion criteria**

Patients initiating MTX (table 1) with at least one of the following criteria:

- Active long-term disease status with ICD-10 code M05 or M06
- At least one hospitalization with an ICD-10 diagnosis code M05 (and subcodes), M06 (and subcodes)

**Exclusion criteria**

Patients with at least one of the following criteria:

- At least one hospitalization or long-term disease with an ICD-10 diagnosis code L93, M32, M34, M60
- Patients with a long-term disease status with a diagnosis of ankylosing spondilytis (ICD-10 M45, M081)
- At least one dispensation of DMARD (including csDMARD) within 3 year prior to MTX initiation, based on ATC codes:
- L01XC02 *Rituximab*
- L04AA13 *Léflunomide*
- L04AA24 *Abatacept*
- L04AA29 *Tofacitinib*
- L04AA37 *Baricitinib*
- L04AB01 *Etanercept*
- L04AB02 *Infliximab*
- L04AB04 *Adalimumab*
- L04AB05 *Certolizumab pegol*
- L04AB06 *Golimumab*
- L04AC03 *Anakinra*
- L04AC07 *Tocilizumab*
- L04AC14 *Sarilumab*

Codes in annex.

Source : (1–5)

# Medical history

Table 3. Identification of medical history over the 5 years preceding the initiation of MTX

**Atherosclerosis**

Patients with at least one hospitalization or long-term disease with an ICD-10 diagnosis code:

- I67.2, I70

**Hypertension**

Patients with at least one of the following criteria:

- at least one hospitalization or long-term disease with an ICD-10 diagnosis code:

I10

I11

I12

I13

I15

- At least 3 reimbusrement at different date for the following treatments, with ATC codes:

C02AB02, C02AC01, C02AC02, C02AC05, C02AC06, C02CA01, C02CA06, C02DC01, C02LA01,

C03AA01, C03AA03, C03BA04, C03BA10, C03BA11, C03BX03, C03CA01, C03CA02, C03CA03, C03DA01, C03DB01, C03EA, C03EA01, C03EA04,

C07AA02, C07AA03, C07AA05, C07AA06, C07AA12, C07AA15, C07AA16, C07AA23,

C07AB02, C07AB03, C07AB04, C07AB05, C07AB07, C07AB08, C07AB12, C07AG01, C07BA02, C07BB02, C07BB03, C07BB07, C07BB12, C07CA03, C07DA06, C07FB02, C07FB03,

C08CA01, C08CA02, C08CA03, C08CA04, C08CA05, C08CA08, C08CA09, C08CA11, C08CA13, C08CX01, C08DA01, C08DB01, C08GA02,

C09AA01, C09AA02, C09AA03, C09AA04, C09AA05, C09AA06, C09AA07, C09AA08, C09AA09, C09AA10, C09AA13, C09AA15, C09AA16, C09BA01, C09BA02, C09BA03, C09BA04, C09BA05, C09BA06, C09BA07, C09BA09, C09BA15, C09BB02, C09BB04, C09BB10, C09BX02, C09CA01, C09CA02, C09CA03, C09CA04, C09CA06, C09CA07, C09CA08, C09DA01, C09DA02, C09DA03, C09DA04, C09DA06, C09DA07, C09DA08,

C09DB01, C09DB02, C09DB04, C09XA02, C09XA52,

C10BX03

**Myocardial infarction**

At least one hospitalization or long-term disease with an ICD-10 diagnosis code:

- I21
- I22
- I23

**Cardiovascular diseases**

At least one hospitalization or long-term disease with an ICD-10 diagnosis code:

- I00 to I99

**Treated dyslipidemia**

At least 3 reimbursement at different date for the following treatments, with ATC codes C10

**Diabetes**

Patients with at least one of the following criteria:

- at least one hospitalization or long-term disease with an ICD-10 diagnosis code:

E10

E11

E12

E13

E14

O24

At least 3 reimbursement at different date for the following treatments, with ATC codes C10 A10

**Hepatic disease**

Patients with at least one of the following criteria:

- Patients with at least one hospitalization or long-term disease with an ICD-10 diagnosis code K70 to K77
- At least 3 reimbursement at different date for the following treatments, with ATC codes:
- J05AB04 *ribavirine*
- J05AE11 *telaprevir*
- J05AE12 *boceprevir*
- J05AE14 *simeprevir*
- J05AF08 *adefovir dipivoxil*
- J05AF10 *entecavir*
- J05AF11 t*elbivudine*
- J05AF05 *lamivudine*
- J05AP08 *sofosbuvir*
- J05AP51 or new code J05AX65 *sofosbuvir and ledipasvir*
- J05AP55 *sofosbuvir + velpatasvir*
- J05AP56 *sofosbuvir and velpatasvir and voxilaprevir*
- J05AP57 *glecaprevir and pibrentasvir*
- J05AX14 *daclatasvir*
- J05AX15 *sofosbuvi*r
- J05AX16 *dasabuvi*r
- J05AX65 *sofosbuvir et ledipasvir*
- J05AX67 *ombitasvir, paritaprevir et ritonavir*
- J05AX68 *elbasvir et grazoprevir*
- At least one biology procedure (NABM classification) for HCV testing:
- 4125
- 4124

**Depression**

Patients with at least one of the following criteria:

Patients with at least one hospitalization or long-term disease with an ICD-10:

- F32
- F33
- F34
- F38
- F39

At least 3 reimbursement at different date for the following treatments, with ATC codes N06A

**Cancer**

Patients with at least one hospitalization or long-term disease with an ICD-10 C00 to C97

**Chronic respiratory disease**

Patients with at least one of the following criteria:

- Patients with at least one hospitalization or long-term disease with an ICD-10:
- I278
- I279
- J40 – J47
- J60 – J67
- J684
- J701
- J703
- At least 2 reimbursement at different date for the following treatments, with ATC codes R03

**Inflammatory bowel disease**

Patients with at least one of the following criteria:

- Patients with at least one hospitalization or long-term disease with an ICD-10:
- K50
- K51
- M07.4
- M07.5
- At least one IBD procedure code CCAM HGQX003

Source : (1,4,5)

.

# Identification of treatment history within 5 years before MTX initiation

Table 4. Identification of treatment history within 5 years before MTX initiation

| **Drug** | **ATC** |
| --- | --- |
| AINS | - M01AB01 *Indométacine* - M01AB05 *Diclofenac* - M01AB16 [*Aceclofenac*](https://en.wikipedia.org/wiki/Aceclofenac) - M01AC01 *Piroxicam* - M01AC02 *Tenoxicam* - M01AC06 *Meloxicam* - M01AE01 *Ibuprofène* - M01AE02 *Naproxène* - M01AE03 *Kétoprofène* - M01AE09 *Flurbiprofène* - M01[AH01](http://www.whocc.no/atc_ddd_index/?code=M01AH01) [*Célécoxib*](https://fr.wikipedia.org/wiki/C%C3%A9l%C3%A9coxib) - M01AX01 [*Nabumétone*](https://en.wikipedia.org/wiki/Nabumetone) - M01[AX02](http://www.whocc.no/atc_ddd_index/?code=M01AX02) [*Acide niflumique*](https://fr.wikipedia.org/wiki/Acide_niflumique) - M01[AE11](http://www.whocc.no/atc_ddd_index/?code=M01AE11) [*Acide tiaprofénique*](https://fr.wikipedia.org/wiki/Acide_tiaprof%C3%A9nique) |
| Analgesics | - N02[AA01](http://www.whocc.no/atc_ddd_index/?code=N02AA01) [*Morphine*](https://fr.wikipedia.org/wiki/Morphine) - N02[AA02](http://www.whocc.no/atc_ddd_index/?code=N02AA02) [*Opium*](https://fr.wikipedia.org/wiki/Opium) - N02AA05 *Oxycodone* - N02AA08 *Dihydrocodéine* - N02AA51 *Morphine, associations* - N02AA55 *Oxycodone, associations* - N02AB03 F*entanyl* - N02AB53 *Fentanyl, associations excluant psycholeptiques* - N02AJ *Opiacés en association avec analgésiques non-opioïdes* - N02AX02 *Tramadol* - N02BE01 *Paracétamol* - N02BE51 *Paracétamol, associations excluant psycholeptiques* |
| Steroids | - H02AB01 *Bétaméthasone* - H02AB02 *Dexaméthasone* - H02AB04 *Méthylprednisolone* - H02AB06 *Prednisolone* - H02AB07 *Prednisone* - H02AB08 Hexacétonide - H02AB09 H*ydrocortisone* |

Sources : (4,6,7)

# Identification of MTX co-dispensations

Table 5. Identification of MTX co-dispensations

| **Drug** | **ATC** |
| --- | --- |
| Sulfasalazine | A07EC01 |
| Acide folique | B03BB01 |
| Rituximab | L01XC02 |
| Léflunomide | L04AA13 |
| Abatacept | L04AA24 |
| Tofacitinib | L04AA29 |
| Baricitinib | L04AA37 |
| Etanercept | L04AB01 |
| Infliximab | L04AB02 |
| Adalimumab | L04AB04 |
| Certolizumab pegol | L04AB05 |
| Golimumab | L04AB06 |
| Anakinra | L04AC03 |
| Tocilizumab | L04AC07 |
| Sarilumab | L04AC14 |
| Aprémilast | L04AA32 |
| Hydroxychloroquine | P01BA02 |

Source : (8) ;

# Identification of patients with a b/tsDMARD initiation over follow-up

Table 5. Identification of patients with a b/tsDMARD initiation over follow-up

| **Drug** | **ATC** |
| --- | --- |
| Rituximab | L01XC02 |
| Abatacept | L04AA24 |
| Tofacitinib | L04AA29 |
| Baricitinib | L04AA37 |
| Etanercept | L04AB01 |
| Infliximab | L04AB02 |
| Adalimumab | L04AB04 |
| Certolizumab pegol | L04AB05 |
| Golimumab | L04AB06 |
| Anakinra | L04AC03 |
| Tocilizumab | L04AC07 |
| Sarilumab | L04AC14 |

# Medical and paramedical visits

Tableau 6. Identification of medical visits

|  | Procedure codes | Speciality code | Settings code |
| --- | --- | --- | --- |
| *General practitioner* | 1111,1112,1113,1114,1115,1117, 1118 | 1, 22, 23 | 51 |
| *Rheumatologist* | 1111,1112,1113,1114,1115,1117, 1118 | 14 | 51 |
| *Dermatologist* | 1111,1112,1113,1114,1115,1117, 1118 | 5 | 51 |

Tableau 7. Identification of paramedical visits

| **Procedure codes** | **Libellé** |
| --- | --- |
| 3111 | AMI |
| 3112 | AIS |
| 3114 | DSI |
| 3115 | Plans de soins |

# Identification des Hospitalizations

Tableau 8. Identification of RA hospitalizations

**Code CIM-10**

M05

M06.0

M06.2

M06.3

M06.8

M06.9

# Identification of imaging procedures of the joints

Tableau 9. Identification of imaging procedures of the joints

| **Code CCAM** | **Libellé** |
| --- | --- |
| Chapitre 13.1.1 | RADIOGRAPHIE DU MEMBRE SUPÉRIEUR |
| Chapitre 13.1.2 | SCANOGRAPHIE DU MEMBRE SUPÉRIEUR |
| Chapitre 13.1.3 | REMNOGRAPHIE [IRM] DU MEMBRE SUPÉRIEUR |
| Chapitre 13.1.6 | ARTHROSCOPIE DU MEMBRE SUPÉRIEUR À VISÉE DIAGNOSTIQUE |
| Chapitre 14.1.1 | ÉCHOGRAPHIE DU MEMBRE INFÉRIEUR |
| Chapitre 14.1.2 | RADIOGRAPHIE DU MEMBRE INFÉRIEUR |
| Chapitre 14.1.3 | SCANOGRAPHIE DU MEMBRE INFÉRIEUR |
| Chapitre 14.1.4 | REMNOGRAPHIE [IRM] DU MEMBRE INFÉRIEUR |
| Chapitre 14.1.7 | ARTHROSCOPIE DU MEMBRE INFÉRIEUR À VISÉE DIAGNOSTIQUE |
| Chapitre 15.1.1 | ÉCHOGRAPHIE DE L'APPAREIL OSTÉOARTICULAIRE ET MUSCULAIRE, SANS PRÉCISION TOPOGRAPHIQUE |
| Chapitre 15.1.2 | RADIOGRAPHIE DE L'APPAREIL OSTÉOARTICULAIRE ET MUSCULAIRE, SANS PRÉCISION TOPOGRAPHIQUE |
| Chapitre 15.1.3 | SCINTIGRAPHIE DE L'APPAREIL OSTÉOARTICULAIRE ET MUSCULAIRE, SANS PRÉCISION TOPOGRAPHIQUE |
| Chapitre 15.1.4 | AUTRES ACTES D'IMAGERIE DE L'APPAREIL OSTÉOARTICULAIRE ET MUSCULAIRE, SANS PRÉCISION TOPOGRAPHIQUE |

# Annexes

**Annex 1. MTX forms by package code**

| **Package code** | **Label** |
| --- | --- |
| **Methotrexate injectable – syringe** | |
| 3400937214216 | METOJECT 10 MG/ML |
| 3400937175432 | METOJECT 10 MG/ML |
| 3400937218238 | METOJECT 10 MG/ML |
| 3400937215626 | METOJECT 10 MG/ML |
| 3400937216975 | METOJECT 10 MG/ML |
| 3400926889050 | METOJECT 10 MG/0.2 ML |
| 3400926774974 | METOJECT 15 MG/0.3 ML |
| 3400926889401 | METOJECT 20 MG/0.4 ML |
| 3400926889920 | METOJECT 25 MG/0,5 ML |
| 3400926888688 | METOJECT 7,5 MG/0.15 ML |
| 3400930016343 | IMETH 10 MG/0,4 ML |
| 3400930016367 | IMETH 12,5 MG/0,5 ML |
| 3400930016381 | IMETH 15 MG/0,6 ML |
| 3400930016404 | IMETH 17,5 MG/0,7 ML |
| 3400930016428 | IMETH 20 MG/0,8 ML |
| 3400930016442 | IMETH 22,5 MG/0,9 ML |
| 3400930016466 | IMETH 25 MG/ML |
| 3400930016312 | IMETH 7,5 MG/0,3 ML |
| 3400930120200 | PREXATE 10 MG/0,40 ML |
| 3400930120231 | PREXATE 12,5 MG/0,31 ML |
| 3400930120842 | PREXATE 15 MG/0,38 ML |
| 3400930120866 | PREXATE 17,5 MG/0,44 ML |
| 3400930120880 | PREXATE 20 MG/0,50 ML |
| 3400930120903 | PREXATE 22,5 MG/0,56 ML |
| 3400930120163 | PREXATE 2,5 MG/0,33 ML |
| 3400930120927 | PREXATE 25 MG/0,63 ML |
| 3400930120941 | PREXATE 27,5 MG/0,69 ML |
| 3400930120972 | PREXATE 30 MG/0,75 ML |
| 3400930120187 | PREXATE 7,5 MG/0,30 ML |
| 3400955745105 | LEDERTREXATE 100 MG/4 ML |
| 3400955813323 | LEDERTREXATE 1000 MG |
| 3400955745044 | LEDERTREXATE 5 000 MG/200 ML |
| 3400931501480 | LEDERTREXATE 50 MG |
| 3400955788676 | LEDERTREXATE 500 MG |
| **Methotrexate injectable – pen** | |
| 3400930020258 | METOJECT 10 MG/0,20 ML |
| 3400930020296 | METOJECT 12,5 MG/0,25 ML |
| 3400930020326 | METOJECT 15 MG/0,30 ML |
| 3400930020364 | METOJECT 17,5 MG/0,35 ML |
| 3400930020395 | METOJECT 20 MG/0,40 ML |
| 3400930020432 | METOJECT 22,5 MG/0,45 ML |
| 3400930020463 | METOJECT 25 MG/0,50 ML |
| 3400930020494 | METOJECT 27,5 MG/0,55 ML |
| 3400930020524 | METOJECT 30 MG/0,60 ML |
| 3400930020197 | METOJECT 7,5 MG/0,15 ML |
| 3400930020463 | METOJECT 25 MG/0,50 ML |
| 3400930075012 | NORDIMET 10 MG |
| 3400930075029 | NORDIMET 12,5 MG |
| 3400930075036 | NORDIMET 15 MG |
| 3400930075043 | NORDIMET 17,5 MG |
| 3400930075067 | NORDIMET 20 MG |
| 3400930075074 | NORDIMET 22,5 MG |
| 3400930075081 | NORDIMET 25 MG |
| 3400930075005 | NORDIMET 7,5 MG |

| **Codes CIP** | **Libellé** |
| --- | --- |
| **Methotrexate oral** | |
| 3400933498269 | NOVATREX 2,5 MG |
| 3400930067840 | NOVATREX 2,5 MG |
| 3400933498030 | NOVATREX 2,5 MG |
| 3400933498320 | NOVATREX 2,5 MG |
| 3400933498498 | NOVATREX 2,5 MG |
| 3400933498559 | NOVATREX 2,5 MG |
| 3400930670682 | METHOTREXATE BELLON 2,5 MG |
| 3400930154182 | METHOTREXATE ACCORD 2,5 mg, comprimé |
| 3400930154137 | METHOTREXATE ACCORD 10 mg, comprimé |
| 3400938974041 | IMETH 10 mg, comprimé sécable |
| 3400957387853 | IMETH 10 mg, comprimé sécable |
| 3400957387914 | IMETH 10 mg, comprimé sécable |
| 3400957388164 | IMETH 10 mg, comprimé sécable |
| 3400957388225 | IMETH 10 mg, comprimé sécable |
| 3400957388393 | IMETH 10 mg, comprimé sécable |
| 3400938983906 | IMETH 2,5 mg, comprimé |
| 3400938984095 | IMETH 2,5 mg, comprimé |
| 3400938984156 | IMETH 2,5 mg, comprimé |
| 3400938984217 | IMETH 2,5 mg, comprimé |
| 3400938984385 | IMETH 2,5 mg, comprimé |
| 3400957391126 | IMETH 2,5 mg, comprimé |
| 3400930004319 | IMETH 2,5 mg, comprimé |
| 3400955001799 | IMETH 2,5 mg, comprimé |

**Annex2. b/tsDMARDs**

| **Codes UCD (rétrocession)** | **Libellé** | **DCI** |
| --- | --- | --- |
| 9434160 | TREMFYA 100MG INJ SRG1ML | Guselkumab |
| 9197702 | MABTHERA 100 mg solution à diluer pour perfusion | Rituximab |
| 9197719 | MABTHERA 500 mg solution à diluer pour perfusion | Rituximab |
| 9423831 | TRUXIMA 500MG PERF FL50ML | Rituximab |
| 9427450 | RIXATHON 100MG PERF FL10ML | Rituximab |
| 9427467 | RIXATHON 500MG PERF FL50ML | Rituximab |
| 9428165 | TRUXIMA 100MG PERF FL10ML | Rituximab |
| 9404377 | ROACTEMRA (TOCILIZUMAB) 162 MG/0,9 ML SOLUTION INJECTABLE EN SERINGUE PREREMPLIE | Tocilizumab |
| 9443791 | ROACTEMRA (TOCILIZUMAB) 162 MG/0,9ML SOLUTION INJECTABLE EN SERINGUE PREREMPLIE | Tocilizumab |

| **Codes UCD (liste en sus)** | **Libellé** | **DCI** |
| --- | --- | --- |
| 9300181 | ORENCIA 250MG PERF FL+SRG | Abatacept |
| 9390267 | ORENCIA 125 MG INJ SRG 1 ML + P/A | Abatacept |
| 9411957 | ORENCIA 125MG INJ STYLO1ML | Abatacept |
| 9447808 | ORENCIA 50MG INJ S.0,4ML 4+P/A | Abatacept |
| 9447814 | ORENCIA 87,5MG INJ S0,7ML4+P/A | Abatacept |
| 9251092 | HUMIRA 40MG INJ SRG0,8ML +T. | Adalimumab |
| 9307562 | HUMIRA 40MG INJ STY0,8ML +T. | Adalimumab |
| 9368664 | HUMIRA 40 MG INJ FL 0,8 ML + NEC | Adalimumab |
| 9416618 | HUMIRA 40MG INJ SRG0,4ML +T. | Adalimumab |
| 9416630 | HUMIRA 40MG INJ STY0,4ML +T. | Adalimumab |
| 9436242 | HUMIRA 20MG INJ SRG0,2ML +T. | Adalimumab |
| 9436259 | HUMIRA 80MG INJ SRG0,8ML +T. | Adalimumab |
| 9436265 | HUMIRA 80MG INJ STY0,8ML +T. | Adalimumab |
| 9438821 | AMGEVITA 20MG INJ SRG0,4ML | Adalimumab |
| 9438838 | AMGEVITA 40MG INJ SRG0,8ML | Adalimumab |
| 9438844 | AMGEVITA 40MG INJ STY0,8ML | Adalimumab |
| 9440019 | IMRALDI 40MG INJ STYL0,8ML | Adalimumab |
| 9440025 | IMRALDI 40MG INJ SRG0,8ML | Adalimumab |
| 9440290 | HYRIMOZ 40MG INJ STYL0,8ML | Adalimumab |
| 9440309 | HYRIMOZ 40MG INJ SRG0,8ML +D | Adalimumab |
| 9441800 | HULIO 40MG INJ SRG0,8ML +T. | Adalimumab |
| 9441817 | HULIO 40MG INJ STYL0,8ML +T. | Adalimumab |
| 9441823 | HULIO 40MG INJ FL0,8ML +NEC | Adalimumab |
| 9450130 | IDACIO 40MG INJ SRG0,8ML +T. | Adalimumab |
| 9450147 | IDACIO 40MG INJ STY0,8ML +T. | Adalimumab |
| 9450153 | IDACIO 40MG INJ FL0,8ML +NEC | Adalimumab |
| 9342601 | CIMZIA 200 MG/ML SOL INJ SER 1ML | Certolizumab pegol |
| 9421223 | CIMZIA 200MG INJ STYLO1ML | Certolizumab pegol |
| 9431032 | CIMZIA 200 MG INJ CART 1 ML | Certolizumab pegol |
| 9242615 | ENBREL 25MG INJ FL+SRG +NEC | Etanercept |
| 9270787 | ENBREL 50MG INJ FL+SRG+NEC | Etanercept |
| 9280024 | ENBREL 50MG INJ FL | Etanercept |
| 9295853 | ENBREL 25MG INJ FL+SRG 0,5ML +NEC | Etanercept |
| 9295876 | ENBREL 25MG/ML PEDIA FL+SRG +NEC | Etanercept |
| 9295882 | ENBREL 50MG INJ SRG 1ML +NEC | Etanercept |
| 9341725 | ENBREL 50MG INJ STY | Etanercept |
| 9391048 | ENBREL 10 MG PEDIA FL + SRG + N | Etanercept |
| 9416653 | BENEPALI 50MG INJ SRG0,98ML | Etanercept |
| 9416676 | BENEPALI 50MG INJ STYLO | Etanercept |
| 9426031 | BENEPALI 25MG INJ SRG0,51ML | Etanercept |
| 9427527 | ERELZI 25MG INJ SRG0.5ML | Etanercept |
| 9427533 | ERELZI 50MG INJ SRG1ML | Etanercept |
| 9427556 | ERELZI 50MG INJ STY1ML | Etanercept |
| 9434467 | ENBREL 25MG INJ STY0,5ML +TAMP | Etanercept |
| 9377120 | SIMPONI 50 MG INJ SRG 0,5 ML | Golimumab |
| 9377137 | SIMPONI 50 MG INJ STY 0,5 ML | Golimumab |
| 9398748 | SIMPONI 100MG INJ SRG1ML | Golimumab |
| 9398754 | SIMPONI 100MG INJ STY1ML | Golimumab |
| 9434160 | TREMFYA 100MG INJ SRG1ML | Guselkumab |
| 9443377 | TREMFYA 100MG INJ STYLO1ML | Guselkumab |
| 9213737 | REMICADE 100MG PERF FL | Infliximab |
| 9398352 | INFLECTRA 100 MG PERF FL | Infliximab |
| 9402303 | REMSIMA 100 MG PERF FL | Infliximab |
| 9418787 | FLIXABI 100MG PERF FL | Infliximab |
| 9438896 | ZESSLY 100MG PERF FL | Infliximab |
| 9000391 | RUXIENCE 100MG PERF FL10ML | Rituximab |
| 9000392 | RUXIENCE 500MG PERF FL50ML | Rituximab |
| 9197702 | MABTHERA 100MG PERF FL10ML | Rituximab |
| 9197719 | MABTHERA 500MG PERF FL50ML | Rituximab |
| 9403691 | MABTHERA 1400 MG INJ SC FL | Rituximab |
| 9423831 | TRUXIMA 500MG PERF FL50ML | Rituximab |
| 9427450 | RIXATHON 100MG PERF FL10ML | Rituximab |
| 9427467 | RIXATHON 500MG PERF FL50ML | Rituximab |
| 9428165 | TRUXIMA 100MG PERF FL10ML | Rituximab |
| 9410604 | COSENTYX 150MG INJ SRG1ML | Secukinumab |
| 9410610 | COSENTYX 150MG INJ STY1ML | Secukinumab |
| 9000261 | ROACTEMRA 20MG/ML INJ FL 10ML (import Irlande) | Tocilizumab |
| 9000262 | ROACTEMRA 20MG/ML INJ FL 20ML (import Irlande) | Tocilizumab |
| 9000263 | ROACTEMRA 20MG/ML INJ FL 4ML (import Irlande) | Tocilizumab |
| 9331879 | ROACTEMRA 20MG/ML INJ FL 10ML | Tocilizumab |
| 9331885 | ROACTEMRA 20MG/ML INJ FL 20ML | Tocilizumab |
| 9331891 | ROACTEMRA 20MG/ML INJ FL 4ML | Tocilizumab |
| 9404377 | ROACTEMRA 162 MG INJ SRG 0,9 ML | Tocilizumab |
| 9443791 | ROACTEMRA 162MG INJ STY0,9ML | Tocilizumab |
| 9328096 | STELARA 45MG INJ FL 0,5ML | Ustekinumab |
| 9351787 | STELARA 45MG INJ SRG 0,5ML | Ustekinumab |
| 9351793 | STELARA 90 MG INJ SRG 1 ML | Ustekinumab |
| 9425020 | STELARA 130MG PERF FL26ML | Ustekinumab |
